# In Vivo Assessment of Elasticity of Child Rib Cortical Bone Using Quantitative Computed Tomography

**DOI:** 10.1155/2017/2471368

**Published:** 2017-07-09

**Authors:** Y. Zhu, F. Bermond, J. Payen de la Garanderie, J.-B. Pialat, B. Sandoz, D. Brizard, J.-P. Pracros, F. Rongieras, W. Skalli, D. Mitton

**Affiliations:** ^1^Université de Lyon, Université Claude Bernard Lyon 1, Ifsttar, LBMC UMR_T9406, 69622 Lyon, France; ^2^School of Automotive Studies, Tongji University, Shanghai 201804, China; ^3^Service de Radiologie, Hôpital Femme Mère Enfant, Lyon, France; ^4^Service de Radiologie, Centre Hospitalier Lyon Sud, Pierre-Bénite, France; ^5^Arts et Metiers ParisTech, LBM/Institut de Biomecanique Humaine Georges Charpak, 151 Bd de l'Hopital, 75013 Paris, France; ^6^Service de Chirurgie Orthopédique et Traumatologique-Hôpital d'Instruction des Armées Desgenettes, 69003 Lyon, France

## Abstract

Elasticity of the child rib cortical bone is poorly known due to the difficulties in obtaining specimens to perform conventional tests. It was shown on the femoral cortical bone that elasticity is strongly correlated with density for both children and adults through a unique relationship. Thus, it is assumed that the relationships between the elasticity and density of adult rib cortical bones could be expanded to include that of children. This study estimated in vivo the elasticity of the child rib cortical bone using quantitative computed tomography (QCT). Twenty-eight children (from 1 to 18 y.o.) were considered. Calibrated QCT images were prescribed for various thoracic pathologies. The Hounsfield units were converted to bone mineral density (BMD). A relationship between the BMD and the elasticity of the rib cortical bone was applied to estimate the elasticity of children's ribs in vivo. The estimated elasticity increases with growth (7.1 ± 2.5 GPa at 1 y.o. up to 11.6 ± 1.9 GPa at 18 y.o.). This data is in agreement with the few previous values obtained using direct measurements. This methodology paves the way for in vivo assessment of the elasticity of the child cortical bone based on calibrated QCT images.

## 1. Introduction

The knowledge of the mechanical properties of the child rib cortical bone could be useful for ribcage models. Such models could be used to assess mechanical loading on the thorax (e.g., for brace treatment or car crash accidents). However, the mechanical properties of pediatric thoracic tissues have been poorly studied due to difficulties in obtaining specimens to perform conventional tests [1, 2]. Regarding specifically the rib cortical bone of children, mechanical data are extremely limited [1]. To the authors' knowledge, only a handful of studies exploring pediatric rib mechanical properties can be found in the existing literature [3–5]. Some other studies did not focus specifically on children, but the population included donors younger than 18 years [6–9].

Compared to the few studies on children's ribs, a significant number of studies performed three-point bending tests on adult rib segments or tensile loading tests on rib coupons to investigate the mechanical properties [9–16], while other studies performed anteroposterior loading tests on the whole ribs [8, 17, 18]. These studies provided detailed knowledge on the human rib mechanical properties of adults. The only existing studies on children's ribs used cadaveric bones or bone tissues collected during surgery but are limited by the number of collected samples. Thus, noninvasive techniques could be extremely valuable to overcome the limitation.

It has been shown previously that variations in trabecular bone properties produced a negligible influence on the mechanical response of a rib model [19]. Therefore, this study focuses on the rib cortical bone. Our group showed recently that quantitative ultrasound can be used to derive rib mechanical properties ex vivo [13], but this technique cannot yet be applied in vivo. It is well known that mechanical properties are related to bone density (physical measurement) [5, 20, 21]. Some studies also showed that the mechanical properties of the femur or tibia bone can be measured by bone mineral density (BMD) using quantitative computed tomography (QCT) [22–26].

The mechanical properties of child and adult cortical bone tissue were found to differ, but the relationship between mechanical properties and ash density is the same for both child and adults [27]. Ash density was previously shown to be strongly related to BMD [25]. Thus, it is assumed that the relationships between elasticity and BMD on the adult rib cortical bone could be expanded to include that of children. Currently, no relationship exists between mechanical properties and BMD of the child rib cortical bone. The current study was designed to fill this gap. Thus, the main goal of this study is to estimate in vivo the elasticity (Young's modulus *E*) of the child rib cortical bone, using calibrated clinical QCT images.

## 2. Materials and Methods

### 2.1. Population

Twenty-eight pediatric patients, including 14 boys and 14 girls, participated in this study. The patients were divided into 7 groups (4 patients per group) according to age (1, 1.5, 3, 6, 10, 15, and 18 years old). All of the children suffered from various known diseases (no rib fractures), and a CT scan was performed on each child's chest for examination purposes (Table 1). This study was determined to be noninterventional by the ethical committee (Sud-Est IV, Lyon, France), and our work was conducted in accordance with the Declaration of Helsinki (1964). The parents were informed of the study, and the images taken were anonymized before analysis in the current study.

### 2.2. CT Scan and Calibration Phantom

All patients' thoracic scans were performed with the same CT scanner (Philips Brilliance 40, Philips, Netherlands). Adaptive tube voltages (80 and 120 kVp) were chosen to minimize the radiation doses regarding the patient corpulence. The matrix maintained a 512 × 512 grid, and the field of view (FOV) was 350 mm × 350 mm, resulting in a pixel size of 0.68 mm^2^ of the CT images. A pad-like calibration phantom (Model 3 CT Calibration Phantom, Mindways Software, USA) was placed on the CT scanner table and under the patient.

### 2.3. BMD Measurement

**Figure 1:**
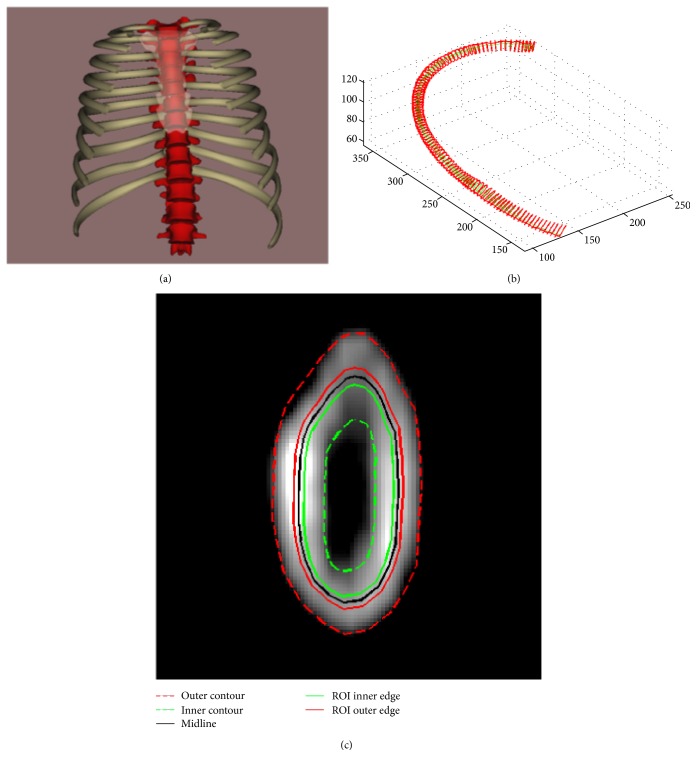
(a) A front view of the 3D reconstructed rib cage from CT slices, (b) an example of 100 equally spaced rib cross-sectional images, and (c) a rib cross-sectional image with contours and ROI.

The CT slices of the rib cage were reconstructed in three dimensions (3D) using a custom software (Figure 1(a)) [28]. For each 6th rib of the patients, 100 equally spaced cross-sectional images from the vertebra to the sternum were extracted using the method previously described by Sandoz et al. [29] (Figure 1(b)). The external and internal contours of the rib cortical bone were also delimited by the software. To ensure the location of the cortical part, the region of interest (ROI) was defined based on the midcontour, which is the center line of the external and internal contours. It was found that assigning 5% to 40% of the inner part of the rib (2.5% to 20% of both sides along the midline) as the region of interest (ROI) gives a stable value of the mean Hounsfield units (HU). To have a conservative approach, we decided to take the 20% inner part (10% of both sides along the midline) as the ROI in this study (Figure 1(c)). The mean HU of the pixels in the ROI was then calculated using MATLAB (MathWorks, Natick, MA, USA). For each studied rib, 10 equally spaced cross-sectional images from 5% to 95% (vertebrae to sternum) of its length were selected to calculate a mean HU. Then, the mean HU was converted into equivalent K_2_HPO_4_ densities (mg K_2_HPO_4_/cm^3^) using the pad-like calibration phantom.

Then, two steps were performed in order to estimate the BMD of child rib cortical bones. First, the Model 3CT calibration Phantom (in mg K_2_HPO_4_/cm^3^, Mindways Software, USA) and the CIRS Phantom 062M (in mg HA/cm^3^, CIRS Inc., Virginia, USA) were imaged simultaneously to establish the relationship between the two units. Three rods of Model 3 CT calibration Phantom (equivalent densities: 58.9, 157, and 375.8 mg K_2_HPO_4_/cm^3^) and three rods of CIRS Phantom 062M (equivalent densities: 200, 800, and 1397 mg HA/cm^3^) were used for calculation. Based on the acquisitions on the 2 phantoms, it was found that the density reported in mg K_2_HPO_4_/cm^3^ was strongly correlated with the density in mg HA/cm^3^ using the following equation, covering cortical bone densities:

(1) $$\begin{array}{c} {BMD}_{clin\;}\left(in\;mg\;HA/{cm}^{3}\right)=1.08\;{BMD}_{clin\;}\left(in\;mg\;K_{2}{HPO}_{4}/{cm}^{3}\right)+55.77\;\left(r^{2}=0.99,SEE=64.26\;mg\;HA/{cm}^{3},p<0.001\right). \end{array}$$

Second, it was found that the BMD measured by clinical QCT (BMD_clin_) was different from that measured by high-resolution peripheral quantitative computed tomography HR-pQCT (BMD_HR_) for a specific rib sample. Indeed, BMD_clin_ was underestimated due to the low resolution of the images. The HR-pQCT scanner provided a clear edge of the rib cortical bone, thus facilitating more accuracy in the measured BMD. To evaluate the influence of the pixel size, 13 adult rib specimens were scanned firstly by clinical QCT (GE LightSpeed Ultra (GE Healthcare, Waukesha, USA), pixel size 0.97 mm × 0.97 mm) calibrated using CIRS Phantom 062M in mg HA/cm^3^ (CIRS Inc., Virginia, USA) and then by HR-pQCT (Xtreme CT, Scanco Medical AG, Brüttisellen, Switzerland, pixel size 0.082 × 0.082 mm^2^). We then provide a regression to convert BMD_clin_ to BMD_HR_.

Based on the 13 adult rib specimens measured by clinical QCT and HR-pQCT (Table 2), it was found that the BMD_clin_ was significantly underestimated compared to the BMD_HR_. For each specific rib specimen, the BMD_clin_ was different but linearly related to the BMD_HR_. According to the analysis methods proposed by Bland and Altman [30], the mean difference between the BMD_clin_ and the BMD_HR_ was 268.12 mg HA/cm^3^ (SD = 67.53 mg HA/cm^3^). The relationship between the BMD measured by these two modalities is as follows:

(2) $$\begin{array}{c} {BMD}_{HR\;}\left(in\;mg\;HA/{cm}^{3}\right)=1.02\;{BMD}_{clin}\left(in\;mg\;HA/{cm}^{3}\right)+258.07\;\left(r^{2}=0.75,SEE=62.09\;mg\;HA/{cm}^{3},p<0.001\right). \end{array}$$

### 2.4. Estimation of the Elasticity

The elasticity (*E*) of the child rib cortical bones was estimated based on the regression presented in [31] on the adult rib (cortical bone) and recalled below:

(3) $$\begin{array}{c} E\;\left(in\;Gpa\right)=0.0143\;{BMD}_{HR\;}\left(in\;mg\;HA/{cm}^{3}\right)-\;2.1768\;\left(r^{2}=0.67,SEE=1.2\;GPa,p<0.01\right), \end{array}$$

where BMD_HR_ was measured by HR-pQCT and reported in mg HA/cm^3^ and the elasticity was assessed using three-point bending tests and an inverse approach [13]. The data for these adult ribs are given in Table 2.

The whole ribs were divided into three parts: the posterior part was defined from 5% to 25% of the rib length, the lateral part from 35% to 65% rib length, and the anterior part from 75% to 95% rib length. In addition to the whole rib, the estimated Young's modulus of child ribs from quantitative computed tomography was assessed for these three parts (posterior, lateral, and anterior).

**Figure 2:**
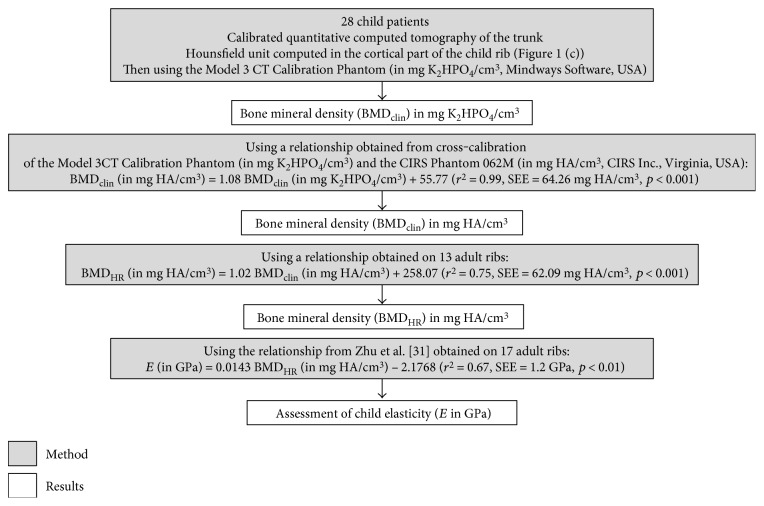
Summary of the methods and results from the quantitative computed tomography acquisition up to the elasticity assessment. In the regressions, SEE stands for standard error of estimate.

Figure 2 illustrates the summary of the methods proposed to estimate the elasticity of child ribs from the calibrated QCT images.

The standard error of estimate (SEE) from each regression was combined to quantify the accumulation of error from the three successive regressions used to derive the cortical bone elasticity. The relative uncertainty on the elasticity was computed as the square root of the sum of the square relative uncertainties (expressed as ax + b + SEE) from the three regressions. The measurement uncertainty on the initial measure of the density BMD_clin_ is ±0.9 mg K_2_HPO_4_/m^3^ and is negligible compared to the uncertainty introduced by the three successive regressions.

A validation of the methodology was conducted by a leave-one-out procedure on adult cortical bone specimens. Elasticity was measured on 13 samples using an inverse approach and 3-point bending experiments (considered the known properties). The same samples were imaged to get their bone mineral density (BMD). One sample out of the 13 was removed to compute a new regression between BMD and elasticity (“regression *n*”). The BMD of this sample was used to predict the bone elasticity using “regression *n*”. The predicted elasticity was compared to the real elasticity obtained from the inverse approach and the 3-point bending experiments. The same process was applied to the 13 samples successively.

### 2.5. Statistics

The statistical analysis was performed using Statgraphics software (version 16.2.04, StatPoint Technologies, Warrenton, USA). The mean and the standard deviation (SD) of the parameters were calculated. Determination coefficient *r*^2^ and standard error of estimate (SEE) were reported to evaluate the correlations.

## 3. Results

The BMD of the child rib cortical bones was assessed and reported in mg HA/cm^3^ based on (1) and (2). The elasticity (*E*) was then estimated based on (3). The combination of the three standard errors of estimate led to an uncertainty of 1.76 GPa compared to a standard error of estimate of 1.2 GPa for (3) alone. The leave-one-out procedure leads to a mean (SD) between the predicted and the real elasticity measurements on adult specimens of the rib cortical bone of −0.18 GPa (2.0 GPa).

The mean HU, BMD_clin_ in mg K_2_HPO_4_/cm^3^, BMD_clin_ in mg HA/cm^3^, BMD_HR_ in mg HA/cm^3^, and elasticity of child ribs in GPa are reported in Table 1.

In Table 3, the estimated Young's modulus of child ribs from quantitative computed tomography along the rib (*E*, *E*_posterior_, *E*_lateral_, and *E*_anterior_) is listed according to the 7 groups.

**Figure 3:**
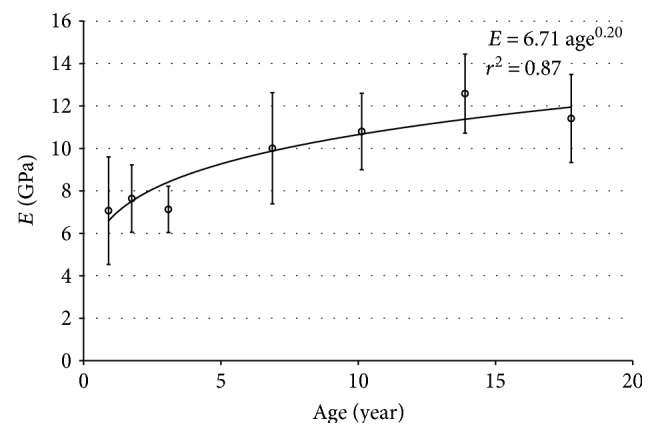
The estimated Young's modulus (*E*) along age.

It was found that the estimated elasticity of the child ribs increased and then decreased from the vertebrae to the sternum, resulting in higher estimated elasticity in the lateral part than in the anterior and the posterior parts. In addition, the mean values of the estimated Young's modulus in each age group were calculated and showed a growing trend with age, as shown in Figure 3. The corresponding regression is as follows:

(4) $$\begin{array}{c} E\;\left(in\;GPa\right)=6.71\;{age}^{0.20\;}\left(age:age\;of\;the\;subject\;in\;year\right)\left(r^{2}=0.87,p<0.001\right). \end{array}$$

## 4. Discussion

The mechanical properties of the child rib cortical bone are poorly known due to the difficulties in obtaining specimens for direct measurements. This study overcame this drawback and proposed a methodology allowing the elasticity of pediatric rib cortical bones to be assessed in vivo. The estimated Young's modulus *E* of pediatric rib cortical bones was computed from a relationship between BMD and elasticity assessed on the adult rib cortical bone. This is the first study which has estimated the elasticity of pediatric rib cortical bones using calibrated QCT images collected clinically.

The proposed methodology is based on the fact that BMD was shown to be a good predictor of elasticity for human cortical bones, assessed by either clinical QCT [22] or HR-pQCT [31]. HR-pQCT can provide high-quality images with clear cortical edges, resulting in accurate BMD measurements. Clinical CT does not have enough resolution to show the edge of the rib cortical bone, which has also been mentioned in the previous studies [32–34]. However, HR-pQCT can only be used for measurements on small-dimension specimens and cannot be used for pediatric thoracic scans. Currently, clinical QCT for BMD measurements is the proper method for in vivo studies. In the current study, the 13 adult rib segments which were scanned in sequence by clinical QCT and HR-pQCT showed that the BMD measured by clinical QCT was significantly underestimated by an average value of 268.12 mg HA/cm^3^. Despite the underestimation of the BMD by clinical QCT, it was proved to be linearly related to the BMD measured by HR-pQCT.

**Figure 4:**
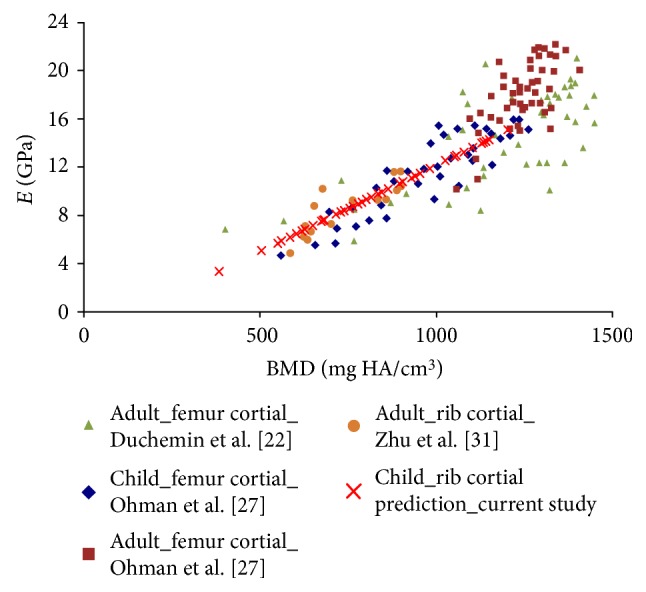
Young's modulus and BMD relationships of human cortical bones, derived from Duchemin et al. [22] and Öhman et al. [27]. The original data were ash densities and were converted into QCT density using the relationship between QCT density and ash density proposed by Kaneko et al. [25], Zhu et al. [31], and the current study.

The mechanical properties of child and adult cortical bone tissue were found to differ, but the relationship between mechanical properties and ash density is the same for both child and adults [27]. The relationship between elasticity and the BMD was found to be similar for the ribs and femurs from children and adults (Figure 4). These results evoke confidence in the assumption we made regarding the assessment of child rib elasticity from a relationship established with adult ribs. In addition, it was found in a recent study [35] that the pediatric cortical bone attained density close to the peak values found in adults a few weeks after birth.

A linear relationship between elasticity and the BMD was used in the current study on cortical bones. In a literature review, Helgason et al. reported nonlinear relationships with cancellous bone [23]. In the density range of the current study, the linear approximation seems reasonable (Figure 4).

**Figure 5:**
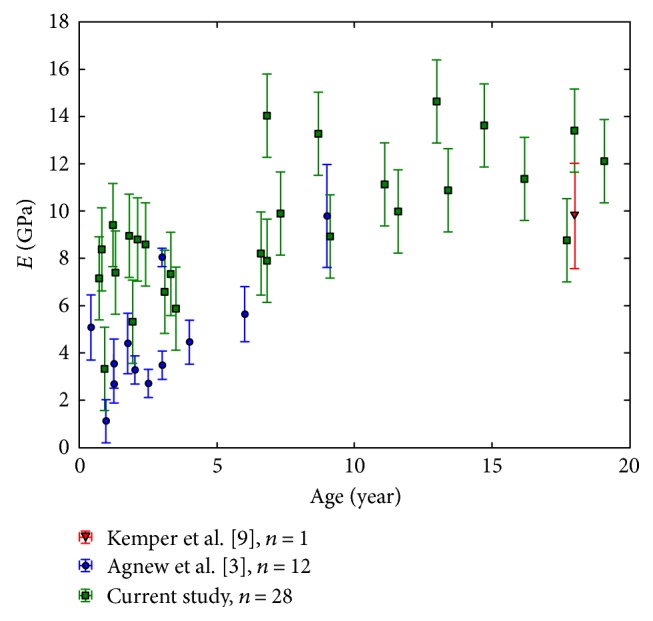
Young's modulus along the age for pediatric ribs from Kemper et al. [9], Agnew et al. [3], and the current study.

To confirm the validity of the current results, a comparison was made with the few direct measurements, in the literature, for the elasticity of pediatric rib cortical bones. Among them, Berteau et al. [4] determined the rib elasticity (Young's modulus in transversal direction) of two teenage subjects (aged 15 and 17 years) using ultrasonic measurements, but due to the difference in orientation, this data cannot be compared to that of the current study. Dynamic tensile tests (strain rate 0.5/s) have been performed on cortical bone coupons harvested from the anterior, lateral, and posterior regions of the ribs [9]. This study on adult specimens included one 18-year-old subject. The mean Young's modulus of 10.0 GPa for that subject is very similar to that obtained in the current study (Figure 5). In another study, quasi-static (loading speed 0.042 mm/s) three-point bending tests on 44 rib sections collected from 12 subjects aged from 5 months to 9 years were performed [3]. They reported a mean Young's modulus of 4.9 GPa, which is similar to and in the lower range of the current results (Figure 5). The lower values obtained in the latter study might be slightly affected by the loading speed. These comparisons suggest that the rib elasticity estimated from quantitative computed tomography images is close to direct measurements backing up the proposed method.

In the current study, the estimated elasticity was found to vary along the rib, from the vertebrae to the sternum, resulting in higher mechanical values in the lateral part compared to the anterior and posterior parts. Similar results have been reported by Stitzel et al. [14] on adult ribs. They reported that the Young's modulus in the lateral portion (11.9 GPa) of the ribs was significantly larger than that in the anterior (7.5 GPa, *p* < 0.001) and posterior (10.7 GPa, *p* < 0.01) parts.

Relationships between estimated elasticity and age were found to be statistically significant. These relationships could be pertinent for studies developing child models (e.g., scaling techniques or parametric studies).

Some limitations should be discussed. Firstly, the elasticity was estimated using the BMD. The pediatric bone development was not considered and may have an effect on the material properties and radioopacity. Even if the pediatric cortical bone attained density close to the peak values found in adults a few weeks after birth [35], BMD alone does not account for the ductile behavior of the child bones compared to the more brittle behavior of the adult bones. BMD is only one of the determinants of the bone elasticity but is the major determinant of the elasticity. Despite the uncertainty of the BMD measurements by clinical QCT, the clinically measured BMD is still the only means by which to estimate the in vivo elasticity for a child population. Using this major determinant of bone elasticity, the estimations of elasticity are consistent with direct measurements of elasticity with child rib cortical bone samples. Secondly, the estimated elasticities from the QCT images were compared to a few direct measurements. Additional direct measurements using child samples would be informative to confirm these promising results. Lastly, the elasticity results should be considered with the uncertainty of the estimation which is 1.76 GPa resulting from the three successive regressions to estimate the elasticity from the calibrated QCT images. This uncertainty could be used for sensitivity studies using rib models. Furthermore, the anisotropy neglected in the current study could be considered in sensitivity studies. The current data should be useful to study the sensitivity of the elasticity in rib models to complete the geometrical sensitivity recently performed [33].

## 5. Conclusion

This study is the first to estimate the elasticity of pediatric rib cortical bones in vivo. Young's modulus was estimated for subjects aged from 1 to 18 years using the calibrated QCT images collected clinically. The elasticity values estimated from the QCT images are similar to those from the direct measurements on pediatric samples. The estimated elasticity of the rib cortical bone increases with age (from 1 to 18 years old). This data can be useful for sensitivity studies using rib models. This noninvasive method overcame the drawback of the paucity of pediatric tissues for biomechanical research. This study paves the way for the estimation of mechanical properties of pediatric cortical bones in vivo.

## Figures and Tables

**Table 1:** Data of the child subjects.

Group	*N*	Subject	Age (year)	Sex	Stature (cm)	Mass (kg)	Description of the disease	Mean HU	BMD_clin_ (mg K_2_HPO_4_/cm^3^)	BMD_clin_ (mg HA/cm^3^)	BMD_HR_ (mg HA/cm^3^)	*E* (GPa)
1	1	DE01	0.9	M	68	7.0	Dyspnea	295.07	63.31	124.14	384.69	3.33
	2	DE02	0.7	M	70	8.6	Cough	675.08	305.53	385.74	651.52	7.14
	3	DE03	0.8	M	80	7.0	Tuberculosis	603.87	383.63	470.09	737.56	8.37
	4	DE04	1.2	F	73	8.0	Pulmonary abscess	883.78	449.22	540.93	809.82	9.41

2	5	DE05	1.8	F	79	10.0	Cystic fibrosis	899.20	420.49	509.90	778.16	8.95
	6	DE06	1.9	F	75	12.0	Neuroblastoma	343.64	190.08	261.06	524.35	5.32
	7	DE07	1.3	F	72	8.0	Cystic fibrosis	697.15	322.18	403.72	669.87	7.40
	8	DE08	2.1	M	90	13.0	Cat scratch	745.84	411.43	500.12	768.19	8.81

3	9	DE09	3.3	F	97	17.5	Chronic bronchitis	476.03	319.95	401.32	667.41	7.37
	10	DE10	3.1	M	90	12.0	Severe asthma	635.31	270.58	347.99	613.02	6.59
	11	DE11	3.5	M	85	13.0	Syndrome	496.98	226.94	300.87	564.96	5.90
	12	DE12	2.4	F	90	13.0	Cystic fibrosis	733.51	398.27	485.90	753.69	8.60

4	13	DE13	6.8	M	122	22.0	Cystic fibrosis	503.93	355.19	439.37	706.23	7.92
	14	DE14	7.3	M	130	27.0	Asthma	705.69	481.37	575.65	845.23	9.91
	15	DE15	6.8	F	118	19.4	Cystic fibrosis	986.13	742.33	857.49	1132.71	14.03
	16	DE16	6.6	M	121	25.0	Pneumonia	520.35	373.05	458.66	725.91	8.21

5	17	DE17	8.7	F	133	27.0	Asthma	926.10	693.71	804.98	1079.14	13.26
	18	DE18	11.1	M	135	50.0	Pneumonia	770.09	557.40	657.76	928.99	11.11
	19	DE19	11.6	F	157	46.0	Pneumonia	616.48	484.89	579.46	849.11	9.97
	20	DE20	9.1	F	140	47.0	Tuberculosis	550.49	419.22	508.53	776.77	8.93

6	21	DE21	14.7	M	170	54.0	Mac lead	917.82	717.03	830.16	1104.84	13.63
	22	DE22	13.4	M	154	50.0	Pneumonia	740.04	543.07	642.29	913.21	10.89
	23	DE23	13.0	F	162	43.0	Lymphedema	898.40	781.26	899.53	1175.59	14.64
	24	DE24	14.4	F	160	53.0	Pneumonia	672.01	562.33	663.08	934.41	11.19

7	25	DE25	18.0	M	180	55.0	Pneumonia	914.38	704.15	816.26	1090.65	13.42
	26	DE26	16.2	F	162	51.0	Pneumonia	676.40	573.82	675.50	947.08	11.37
	27	DE27	17.7	M	180	82.0	Lymphedema	615.38	408.89	497.37	765.39	8.77
	28	DE28	19.1	F	181	73.0	Teratoma	792.04	620.10	725.48	998.06	12.10

		Mean	7.8		120	30.5		688.97	456.41	548.69	817.74	9.52
		SD	6.1		39	22.0		178.37	177.30	191.49	195.32	2.79

**Table 2:** Characteristics of the adult cadaveric specimens. Bone mineral density measured by quantitative computed tomography (BMD_clin_); bone mineral density measured by high-resolution peripheral computed tomography (BMD_HR_); the Young's modulus (*E*_inv_) comes from [13] using three-point bending tests and an inverse approach.

*N*	Specimen ID	Age (years)	Sex	BMD_clin_ (mg HA/cm^3^)	BMD_HR_ (mg HA/cm^3^)	*E*_inv_ (GPa)
1	67-2010_6R	85	M	357.3	623.4	4.9
2	67-2010_8R	85	M	521.0	729.2	8.8
3	206-2010_6L	67	M	515.2	636.7	6.3
4	206-2010_8L	67	M	555.8	823.3	7.4
5	211-2010_6L	80	M	535.9	897.8	11.7
6	211-2010_8L	80	M	673.3	878.9	11.6
7	250-2010_6L	80	M	631.5	901.2	10.4
8	250-2010_8R	80	M	672.9	889.0	10.1
9	24-2011_6R	65	M	505.3	836.4	9.3
10	138-2011_6L	77	M	302.8	633.3	6.0
11	138-2011_8L	77	M	290.0	633.4	6.9
12	144-2011_6L	82	F	407.2	677.0	6.7
13	144-2011_8L	82	F	349.9	644.2	10.2

Mean		76.6		486.0	754.1	8.5
SD		7.6		133.6	117.7	2.3

**Table 3:** Estimated Young's modulus of child ribs from quantitative computed tomography along the rib.

Group		Age (year)	*E* ^1^	*E*_posterior_^2^	*E*_lateral_^3^	*E*_anterior_^4^
1	Mean	0.9	7.1	6.8	8.0	6.5
	SD	0.2	2.5	1.8	2.9	2.6

2	Mean	1.8	7.6	6.2	8.7	7.9
	SD	0.4	1.6	1.0	2.4	2.4

3	Mean	3.1	7.1	6.6	8.0	6.6
	SD	0.5	1.1	1.2	1.5	1.7

4	Mean	6.9	10.0	8.2	11.2	9.6
	SD	0.3	2.6	2.2	3.3	3.0

5	Mean	10.1	10.8	10.9	11.5	9.8
	SD	1.3	1.8	1.3	2.0	2.8

6	Mean	13.9	12.6	11.7	14.4	11.2
	SD	0.7	1.9	1.8	2.7	2.1

7	Mean	17.8	11.6	9.3	13.0	11.3
	SD	1.1	1.9	2.7	3.2	1.6

^1^Global: slices 5%–95%; ^2^posterior: slices 5%–25%; ^3^lateral: slices 35%–65%; ^4^anterior: slices 75%–95%.
